# Incarcerated Lumbar Hernia Complicated by Retroperitoneal Pseudoaneurysm 50 Years after Resection and Radiation Therapy of a Sarcoma

**DOI:** 10.1155/2019/1072821

**Published:** 2019-04-28

**Authors:** Oluwatobi Onafowokan, Dabanjan Bandyopadhyay, Dale Johnson, Hugo J. R. Bonatti

**Affiliations:** ^1^University of Maryland Community Medical Group, Easton, MD, USA; ^2^Meritus Surgical Specialists, Hagerstown, MD, USA

## Abstract

**Background:**

Lumbar hernias are rare abdominal hernias. Surgery is the only treatment option but remains challenging. Posterior incisional hernias are even rarer especially with incarceration of intra-abdominal contents.

**Case Presentation:**

A 68-year old female presented with a 3-day history of worsening acute abdominal pain and distension, with multiple episodes of emesis. A CT scan indicated a large incarcerated posterolateral abdominal hernia. The patient had a history of resection of a sarcoma on her back as a child and also received chemotherapy and radiation. During emergency laparoscopy, a hemorrhagic small bowel segment incarcerated in the hernia was reduced and resected, and the distended small bowel was decompressed. An elective hernia repair was scheduled. After temporary clinical improvement, the patient again developed abdominal pain, distention, and emesis. During emergency laparotomy, a large hematoma in the right flank was found and partially evacuated. The right colon was mobilized out of the hernia and the duodenum was kocherized. A 20 × 20 cm BIO-A mesh was placed on top of the Gerota fascia and cranially tucked under liver segment VI. Anteriorly, the mesh was fixated with absorbable tacks. The duodenum and colon were placed into the mesh pocket. A postoperative CT scan identified a 2 cm pseudoaneurysm of a side branch of a lumbar artery, and the bleeding source was embolized. The postoperative course was complicated by *Clostridium difficile*-associated colitis, but ultimately, the patient recovered fully. At 6-month follow-up, there was no evidence for a recurrent hernia.

**Discussion:**

There is a paucity of literature concerning lumbar incisional hernias. Repair with bioabsorbable mesh seems feasible, but longer follow-up is necessary as the mesh was placed in an unusual fashion due to the retroperitoneal hematoma. The exact cause of the hemorrhage is unclear and may have been caused during the initial incarceration, during surgery, or may be a late complication of her previous radiation.

## 1. Introduction

Lumbar hernias develop through defects in the posterior and/or posterolateral abdominal wall and may contain abdominal organs [1–4]. They may be congenital, posttraumatic, or occur following surgery of retroperitoneal organs [2, 4, 5]. Lumbar hernias typically occur in two established weak areas of the posterolateral abdominal wall. The superior lumbar triangle of Grynfeltt is more commonly involved than the inferior lumbar triangle of Petit [2, 6–9]. Acquired posterolateral hernias are now much more common than hernias at the lumbar triangles [10]. Affected individuals may present with back pain, a back mass, bowel obstruction, or urinary obstruction if the kidney or ureters become entrapped in the hernia [1, 3, 4]. Surgical repair may be difficult due to the surrounding tissues being either bone or other tissue which may be difficult to approximate and may not adequately support a mesh used for repair [2, 3].

Older publications described primary closure with suturing of the defect and this was later replaced by open surgery reinforcing the defect using mesh [1]. Most recently, the laparoscopic approach has gained popularity; however, no randomized or controlled studies are available. For lumbar incisional hernias, it seems that frequently the decision on the surgical approach is based on the individual situation and also depends on acuity of the case and patient demographics, size, and localisation of the hernia as well as surgeon preference and experience [10]. A limited number of cases (~300) have been reported with first descriptions dating back almost 400 years [6].

We report the clinical course of on an elderly female who presented with an incarcerated incisional posterior abdominal wall hernia and had an unusual clinical course.

## 2. Case Presentation

A 68-year-old female presented to the emergency room with a 3-day history of acute abdominal pain, nausea, and emesis. She had not felt well for several weeks with poor appetite, intermittent abdominal cramping, and right flank pain. Significant comorbid conditions included hypertension and hyperlipidaemia; she was an active smoker. Medical history included abdominal hysterectomy, and as a child, she underwent lumbar surgery with resection of a right paravertebral sarcoma, for which she also received chemotherapy and radiation. No medical records for this disease were available and the patient could not give additional information. As a sequela of her treatment, she had a large bulge in her right flank with significant soft tissue swelling and telangiectatic skin. She knew about the lumbar hernia, which developed few years after her surgery, but she was never symptomatic and did not desire repair.

**Figure 1:**
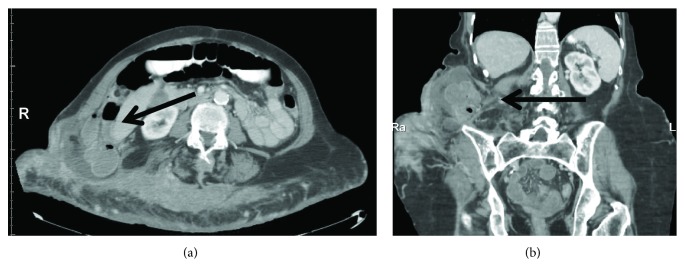
(a, b) CT scan. Bowel herniation through the right posterolateral abdominal wall (arrows).

On examination, she was tachycardic and short of breath. Her abdomen was significantly distended and tender, and there was a large bulge noted in her right flank. Her white blood count was elevated at 14 K/microL, serum sodium (132 mEq/L) and chloride (93 mEq/L) were low, and creatinine was borderline elevated (1.7 mg/dL), reflecting dehydration. Pain control was achieved and she was resuscitated with 2 liters of normal saline while oral contrast was given. CT scan showed a large, incarcerated, posterolateral abdominal hernia with free fluid and possible free air and distended bowel loops indicative of obstruction (Figures 1(a) and 1(b)). A nasogastric tube was placed and 1500 mL of small bowel content was evacuated and she was posted for emergency surgery.

**Figure 2:**
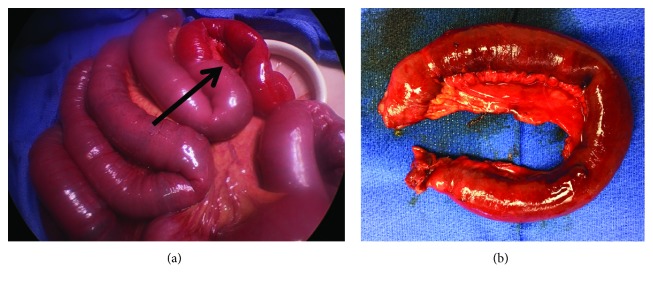
(a, b) Operative findings. Hemorrhagic small bowel segment (arrow).

**Figure 3:**
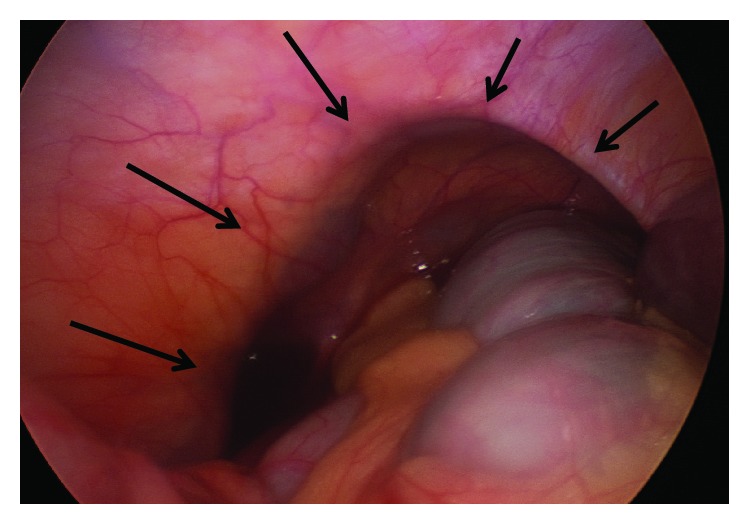
Laparoscopic findings. Large lumbar hernia (arrows) with the colon trapped inside.

She was placed supine and the abdomen was accessed with a 5 mm Kii Fios first entry port (Applied Medical, Rancho Santa Margarita, CA) in the left upper quadrant (LUQ). Pneumoperitoneum was established and a second 5 mm trocar was placed under visual control in the left lower quadrant (LLQ). Multiple distended small bowel loops and a distended ascending and transverse colon were visualized. Superior to the proximal transverse colon and lateral and inferior to the liver, the roof of the hernia with the hepatic flexure trapped inside was seen. The small bowel segment trapped inside the hernia was reduced and found to be hemorrhagic but there was no perforation. The small bowel was eviscerated from a 4 cm minilaparotomy using her old subumbilical midline incision, and the hemorrhagic loop did not recover and seemed unsalvageable (Figure 2(a)). The small bowel distally was collapsed. The healthy segments proximal and distal to the damaged loop were aligned, hold stitches were placed, and through the enterotomies for the linear stapler, the proximal small bowel was decompressed. The anastomosis was created using a GIA-75 stapler, and the damaged small bowel loop (Figure 2(b)) was resected together with the common enterotomy with a second transverse staple load. Pathology confirmed extensive hemorrhagic necrosis of the mucosa extending in some areas into the muscle layer. The bowel was placed back into the abdomen, the minilaparotomy was closed, and pneumoperitoneum was reestablished. Visualization was now much improved. The anastomosis was laid in the RUQ. The hernia was noted to be large, with the hepatic flexure partially trapped in it (Figure 3). Decision was made not to repair the hernia at this stage due to the contamination from the damaged small bowel loop, but laparoscopic hernia closure using a mesh was planned to take place within few days.

Three days later, the patient complained of increasing abdominal distension and abdominal and right flank pain. X-ray showed increased distension of small bowel loops and recurrent incarceration of bowel was suspected. At emergency laparotomy through extension of the previous incision cranially and caudally, significantly dilated small bowel loops, transverse colon, and right hemicolon were noted. These were decompressed through the nasogastric tube but no bowel was incarcerated within the hernia. A large hematoma was found in the right flank, lateral and dorsal to the ascending colon. The white line of Toldt was opened, and a portion of the hematoma was carefully suctioned out, and a pad was placed to tamponade the retroperitoneum. No acute bleeding source was identified. The ascending and transverse colon were mobilized out of the hernia. The duodenum was kocherized and the Gerota fascia was exposed. We opted not to mobilize the right kidney due to fear that a major retroperitoneal bleed could be caused from the source that had fed the hematoma and was now tamponaded. The hernia borders could be determined but no primary closure was possible. Therefore, the defect was covered using a 20 × 20 cm BIO-A mesh (Gore, Flagstaff, AZ). The mesh was placed laterally and dorsally onto the Gerota fascia as the kidney was covering this portion of the hernia and tucked under liver segment 6. Absorbable tacks were used to secure the mesh into the anterior and lateral abdominal wall. The duodenum and right colon were placed into the created mesh pocket. A 19-Blake drain was placed on top of the mesh, next to the colon.

**Figure 4:**
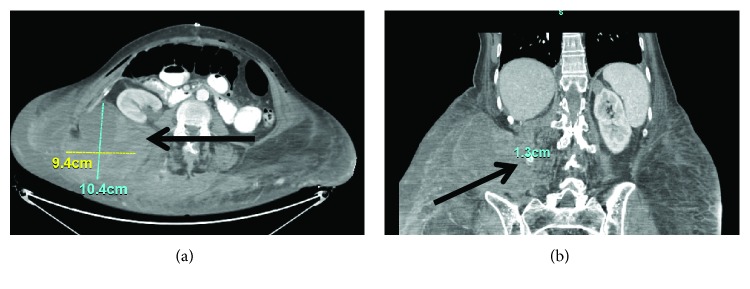
(a, b) CT. Right-sided retroperitoneal hematoma with pseudoaneurysm (arrows).

**Figure 5:**
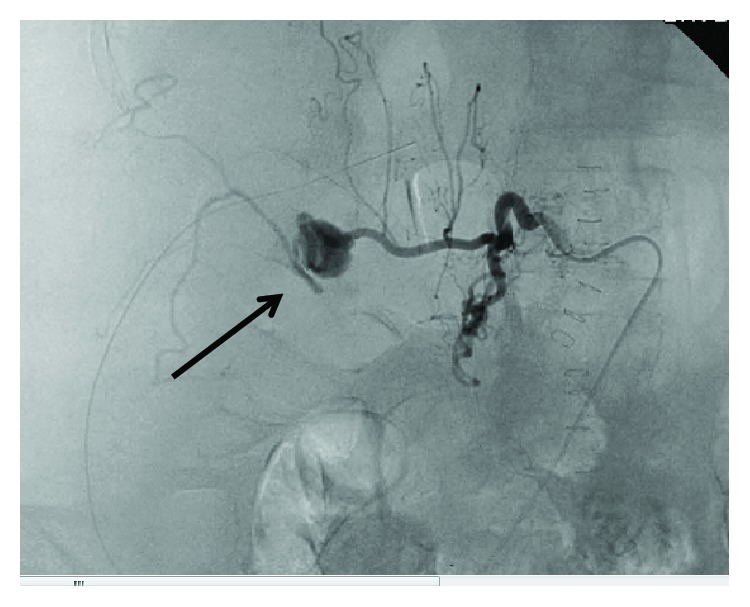
Angiography. Lumbar pseudoaneurysm (arrows).

**Figure 6:**
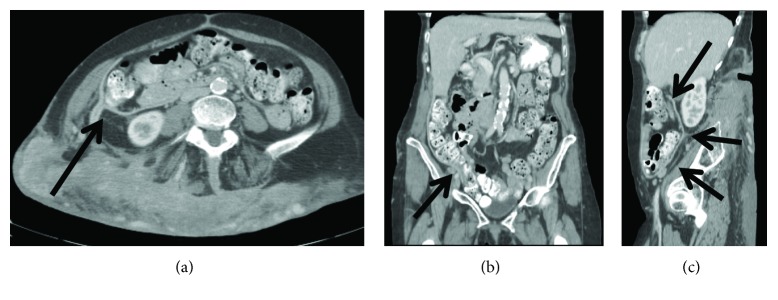
(a–c) Follow-up CT scan (3 planes) at 6 months. Hernia covered by Bio-A mesh (arrows).

CT scan the next day identified a 2 cm pseudoaneurysm of a side branch of a lumbar artery as source of the hematoma (Figures 4(a) and 4(b)). This was subsequently embolized by interventional radiology (Figure 5). The postoperative course was complicated by *Clostridium difficile*-associated colitis, but ultimately, the patient recovered fully. At 6-month follow-up, she had gained weight, had continued to refrain from smoking, and had no evidence for recurrent hernia. On CT scan, the mesh covered the previous defect (Figures 6(a)–6(c)) and no recurrent hernia developed. Tissue changes in the area of the previous radiation were still visible, and percutaneous biopsy was recommended to exclude development of a secondary malignancy, which the patient at this time declined.

## 3. Discussion

Lumbar hernias may contain intraperitoneal organs such as bowel [11] or be completely extraperitoneal containing retroperitoneal fat and kidneys [12]; less commonly, the ovaries, spleen, or appendix are found inside [13, 14]. Incisional lumbar hernias [15–17] are most often associated with nephrectomy, abdominal wall tumour resection, aortic aneurysm repair, and iliac bone graft harvest [8, 18–22]. Damage to the subcostal nerve and the subsequent muscular atrophy with progressive thinning of muscle and fascia may be an important predisposing factor for the development of incisional posterior hernias [13, 23, 24].

Lumbar hernias gradually enlarge after they form, but patients are often asymptomatic for a long time [2]. Strangulation of abdominal contents occurs in approximately only 10% of cases as the hernia neck typically is wide [25, 26]. Due to their relatively infrequent occurrence, there is no established definitive technique for the surgical repair of lumbar hernias [10]. Anterior repair was considered appropriate for managing large or recurrent defects, with the use of a double mesh or flaps from the aponeurosis of the gluteus maximus muscle [27, 28]. Laparoscopic repair has recently been reported to be effective with transperitoneal and preperitoneal approach both being feasible [29–35].

During her first procedure, we opted for a laparoscopicapproach and damage control with reduction of hernia contents, small bowel decompression, and resection of a hemorrhagic loop and not to repair the hernia based on the contamination and the fact that extensive mobilization using a laparotomy would have been necessary. Rather, we had planned to repair the hernia in a minimal invasive fashion after recovery. We also contemplated if the hernia was in fact congenital and not acquired based on the intraoperative findings.

Open hernia repair with mesh during emergency laparotomy was done similar to the case published by Teo et al. [36]; however, traumatic hernias may be repaired laparoscopically in the elective setting [31, 37–39]. The reported experience with strangulated lumbar hernias is very limited as recently summarized by Ka et al. [40]. In most cases, open surgery is necessary if such an emergent situation develops, especially if entrapped contents become severely damaged. We attempted a staged repair after damage control but were forced to reintervene due to the development of an acute abdomen. To our surprise, a retroperitoneal hematoma and not a repeat incarceration of the bowel was the cause and this was an ileus and not an obstruction. The hematoma, which we believe was contributing to the ileus, was partially evacuated. We were reluctant to completely mobilize the right kidney for placing the mesh on the psoas muscle, which would be the more established approach. For the hernia repair, which may only be a temporary solution, we opted for a biodegradable product. Anchoring of mesh to cover dorsal hernias may be difficult and needs to be adapted to the individual situation [10]. The duodenum and right colon kept the mesh in place as demonstrated by follow-up CT scan after three months. The retroperitoneal pseudoaneurysm, which was identified as the bleeding source, was embolized the next day. It is unclear how the pseudoaneurysm developed, but most likely, a minimal trauma either from the incarceration or during first surgery injured a blood vessel in the area of the previous radiation and low molecular heparin for DVT prophylaxis may have propagated bleeding. Such aneurysms are rare and associated with trauma, retroperitoneal biopsies, and surgery of the lumbar spine amongst others [41]. Bleeding is best controlled using percutaneous embolization such as in our case [41–43]. The patient had a protracted course but ultimately did well. Much longer observation is necessary to determine if the Bio-A mesh placed in this unusual fashion will prevent recurrence.

To summarize, incisional lumbar hernias are rare. Surgical approach needs to be tailored to the individual presentation and a staged approach may be required. A bioabsorbable mesh may be a good option in similar cases.
